# Recombinant DnaK Orally Administered Protects Axenic European Sea Bass Against Vibriosis

**DOI:** 10.3389/fimmu.2019.03162

**Published:** 2020-02-14

**Authors:** Eamy Nursaliza Yaacob, Parisa Norouzitallab, Bruno G. De Geest, Aline Bajek, Kristof Dierckens, Peter Bossier, Daisy Vanrompay

**Affiliations:** ^1^Laboratory of Aquaculture and Artemia Reference Center, Faculty of Bioscience Engineering, Ghent University, Ghent, Belgium; ^2^Laboratory for Immunology and Animal Biotechnology, Faculty of Bioscience Engineering, Ghent University, Ghent, Belgium; ^3^Department of Pharmaceutics, Ghent University, Ghent, Belgium; ^4^Écloserie Marine de Gravelines, Gravelines, France

**Keywords:** gnotobiotic, European sea bass larvae, bacterial HSP70, DnaK, immunity, vibriosis, *Vibrio anguillarum*

## Abstract

*Vibrio anguillarum* causes high mortality in European sea bass (*Dicentrarchus labrax*) larviculture and is a hindering factor for successful sustainable aquaculture of this commercially valuable species. Priming of the innate immune system through administration of immunostimulants has become an important approach to control disease outbreaks in marine fish larviculture. This study was conducted to evaluate immunostimulation by *Escherichia coli* HSP70 (DnaK) in axenic European sea bass larvae in order to protect the larvae against vibriosis. DnaK stimulates the immune response in crustaceans and juvenile fish against bacterial infections. The use of axenic fish larvae allows to study immunostimulation in the absence of an interfering microbial community. At 7 days post-hatching, larvae received a single dose of alginate encapsulated recombinant DnaK. Two non-treated control groups in which animals either received empty alginate microparticles (C1) or no alginante microparticles (C2 and C3) were included in the study. Eighteen hours later, all larvae, except the ones from group C3 (non-infected control) were challenged with *V. anguillarum* (10^5^ CFU, bath infection). Mortality was daily recorded until 120 h post infection and at 18, 24, and 36 h post infection, larvae were sampled for expression of immune related genes. Results showed that *V. anguillarum* induced an immune response in axenic sea bass larvae but that the innate immune response was incapable to protect the larvae against deadly septicaemic disease. In addition, we showed that administration of alginate encapsulated DnaK to axenic European sea bass larvae at DAH7 resulted in a significant, DnaK dose dependent, upreglation of immune sensor, regulatory and effector genes. Significant upregulation of *cxcr4, cas1* and especially of *hep* and *dic* was correlated with significant higher survival rates in *V. anguillarum* infected larvae. In the future recombinant DnaK might perhaps be used as a novel immunostimulant in sea bass larviculture.

## Introduction

The production of farmed fish has increases 12 times in the past three decades at an average annual growth of 8%, making making it the fastest growing food production sector (1). In Europe, the three major species reared are Atlantic salmon (*Salmo salar*), European sea bass (*Dicentrarchus labrax*) and sea bream (*Sparus aurata*). In order to further support the growth of output, production of fish juveniles for stocking on growing farms has to be increased. However, the latter is hampered by high and unpredictable mortality of fish larvae after hatching (2). Compared to the other cultured fish species, highly sensitive to disease outbreaks. The increased sensitivity of sea bass is related to its strong and prolonged stress-induced cortisol increase and increased biosynthetic capacity of interrenal tissue involving a high turnover in the corticosteroid production and release (3). Sea bass larvae are highly susceptible to the opportunistic pathogen *Vibrio anguillarum* [reviewed in (4)]. *V. anguillarum*, a Gram-negative bacterium, causes a deadly septicaemic disease (5, 6). The skin, gills and intestinal epithelium are replication sites for *Vibrio*. In sea bass, *V. anguillarum* evades the innate immune response by inhibiting the respiratory burst reaction in macrophages and by downregulating the expression of apoptotic caspases (7). In addition, many *Vibrio* strains have siderophore-dependent and/or siderophore-independent iron-acquisition mechanisms [reviewed in (8, 9)], which enables them to use iron for growth even if iron-binding, fish proteins such as ferritin and transferrin are present.

Strategies for preventing vibriosis outbreaks in sea bass larviculture are urgently needed. Preventive use of antibiotics and chemotherapeutics is, for obvious reasons, no longer allowed. Stimulation of the innate immune system [reviewed by (10, 11)] of larvae or maternal transfer of immunity [maternal IgM, lectin, lysozyme, C3, factor B, phosvitin, lipovitellin; reviewed by (12–15)] is an alternative approach. Administration of immunostimulants in larvae is particularly interesting, as they might protect the larvae against vibriosis and at the same time create a broad spectrum innate immune memory (11). Thus, prophylaxis of infectious diseases in fish larviculture currently focuses on the stimulation of the innate immune system either by manipulating the environment for example with elevated temperature (16, 17) or supplying the diet with immunostimulants (18, 19).

Some of the immunostimulants, such as bacterial products (11), and plant constituents (20–24) are considered as the activators of endogenous heat shock proteins (HSPs). Heat shock proteins are a family of evolutionary conserved cellular proteins and they exist in all living organisms. Under normal physiological conditions, these proteins are produced constitutively and play a role in protein metabolism. However, following adverse conditions, the level of these proteins is increased in order to protect cells from lethal damage (12, 25). In the case of disease outbreaks, increased levels of HSPs can activate an innate immune response through a danger-associated molecular pattern (DAMP) cascade. HSP70 is a member of HSPs family with a size of 70KDa. The prokaryotic HSP70 is often referred to as DnaK. Eukaryotic HSP70 and DnaK have been reported to stimulate the immune response in brine shrimp (*Artemia franciscana*), Pacific white shrimp (*Litopenaeus vannamei*) and Southern platyfish (*Xiphophorus maculatus*) against bacterial infections (20, 26–29).

In this study, we examined the effectiveness of recombinant *Escherichia coli* DnaK as an immunostimulant for fish larvae and its potential for future oral mass application in sea bass larviculture for protecting the animals against vibriosis. To address this, a previously validated experimental infection model in gnotobiotic sea bass (*D. labrax*) larvae was used (30). Maintaining sea bass larvae under gnotobiotic conditions allows full control over the host-associated microbial communities (31). Therefore, it represents an exceptional experimental system for carrying out immunological studies related to host-pathogen interactions. The gnotobiotic system eliminates any possible microbial interference during the DnaK exposure period and hence facilitates the interpretation of the results in terms of a cause-effect relationship (32). DnaK was administered orally, encapsulated in alginate microparticles. The method for preparation and characterization of alginate microparticles for oral administration of immunostimulants to gnotobiotic sea bass larvae at day after hatching 7, has previously been established (33). The commercial alginate used is a polysaccharide, extracted from brown algae (kelp). Alginate possesses muco-adhesive properties and is considered as safe due to its biodegradability, its low toxicity and low immunogenicity (34). Alginate microparticles are stable in sea water and they are successfully ingested by sea bass larvae whereafter their content is released in the gut (33).

## Materials and Methods

### Cloning, Production, and Purification of Recombinant Bacterial HSP70 (DnaK)

**Figure 1:**
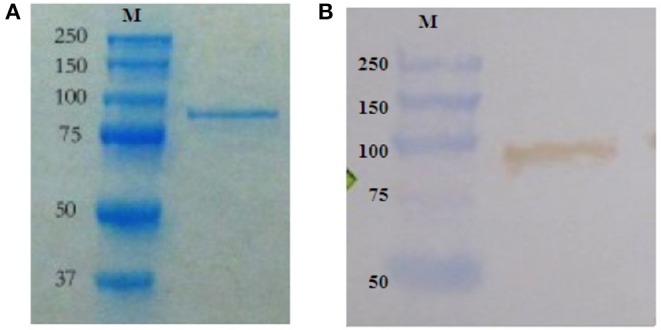
Arabinose-induced over-production of DnaK by the E_native_ *E. coli* strain. Purified recombinant DnaK (83 kDa), with additional amino acid residues being an N-terminal thioredoxin and C-terminal polyhistidine (6 × His) tag, by SDS-PAGE gel electrophoresis and Western blotting. **(A)** Coomassie staining of polyacrylamide gel loaded with purified recombinant DnaK. Molecular weight markers (M) in kDa are on the left. **(B)** PVDF membrane probed for purified recombinant DnaK using a mouse monoclonal antibody against DnaK followed by HRP-labeled donkey anti-mouse IgG (H + L), substrate and chromogen. Molecular weight markers (M) in kDa are on the left.

Recombinant DnaK (83 kDa), with additional amino acid residues being an N-terminal thioredoxin and C-terminal polyhistidine (6 × His) tag, was expressed by E_native_, an *E. coli* strain (MG155; accession number: AIZ92815.1) over-expressing recombinant DnaK (28, 29) was cultured at 37°C in Luria Bertani (LB) broth containing 50 μg ml^−1^ kanamycin (Sigma-Aldrich, Diegem, Belgium). Over-expression of DnaK was induced by adding 0.5 mg/ml^−1^ of arabinose (Sigma-Aldrich) to the culture and incubation for 4 h at 37°C. For bacterial cell disruption and DnaK collection, cultured bacteria were transferred to sterile vials in which sterile glass beads (0.1 mm) were added. The vials were then placed in a bead beater (Lab Services, Breda, The Netherlands) and the cell wall of the bacteria was mechanically disrupted by agitation (twice for 30 s). The bacterial lysate was collected by centrifugation (2200 x *g* for 1 min at 4°C) and the subsequent purification of DnaK was performed using a Dynabeads® His-Tag Isolation & Pulldown kit (Life Technologies, Aalst, Belgium) following the manufacturer's protocol. The endotoxin content was determined using the Toxin Sensor Chromogenic LAL Endotoxin Assay Kit (GenScript, New Jersey, USA) following the manufacturer's protocol. Purified DnaK was stored at −80°C. The concentration of the purified DnaK was determined using a Quick Start™ Bradford protein assay (Bio-Rad Laboratories, Temse, Belgium) following the manufacturer's protocol. Samples were analyzed by SDS-PAGE using the procedure previously described (35). Precision Plus Protein™ (Bio-Rad Laboratories) was used as a pre-stained molecular weight marker (MW range 10–180 kDa). Proteins were stained with the Bio-Safe Coomassie stain (Bio-Rad Laboratories) or electrophoretically transferred to a polyvinylidene difluoride (PVDF) membrane (Immun-Blot® PVDF membrane, Bio-Rad Laboratories) using a semi-dry Western blotting system (250 mA, 1 h; Trans-Blot® Semi-Dry, Bio-Rad Laboratories). The PVDF membrane was blocked (PBS with 0.2% Tween-20 and 5% BSA) for 1 h at room temperature (RT) and subsequently incubated overnight (4°C) with a mouse monoclonal antibody against DnaK (8E2/2; diluted 1:5000) (Enzo Life Sciences, Antwerpen, Belgium). The membrane was washed and thereafter incubated with horseradish peroxidase-labeled donkey anti-mouse IgG (H + L) (Affinity BioReagents™, Colorado, USA) at a dilution of 1:2500 (1 h, RT). After washing, 0.7 mM 3,3′-diaminobenzidine (Sigma-Aldrich) and 0.01% H_2_O_2_ in 0.1 M Tris-HCl (pH 7.6) were added (10 min, RT). Membranes were washed and analyzed with Western blot imaging system ChemiDoc™ MP (Bio-Rad Laboratories) (Figure 1).

### Microparticle Preparation and Antigen Encapsulation

Recombinant *E. coli* DnaK was encapsulated in calcium alginate microparticles. Preparation of DnaK loaded microparticles (median size of 84 ± 4.7 μm) for oral administration to gnotobiotic sea bass larvae at day after hatching (DAH) 7, was done as previously described (33). Briefly, three ml of an aqueous sodium alginate solution prepared by using low viscosity alginate (A2158; Sigma-Aldrich, Diegem, Belgium), was dispersed in 12 ml iso-octane (Sigma-Aldrich) solution containing two lipophilic surfactants, 0.05% (v/v) Span-80 (Fagron, Weregem, Belgium) and 0.02% (v/v) Tween-80 (Fagron). The solution was stirred for 3 min using a magnetic stirrer. Subsequently, 1500 μg (500 μg ml^−1^ sodium alginate solution) recombinant DnaK was added to the aqueous sodium alginate solution. The emulsion was homogenized for 2 min using a Turrax homogenizer (Bodart & Co., Antwerp, Belgium). Next, 3 ml of a calcium chloride solution (700 mM; Sigma-Aldrich) was added to the mixture and was stirred for another 2 min. To further harden the formed microparticles, 15 ml of isopropyl alcohol (Fagron) was added. The resulting microparticles were separated and collected by centrifugation (300 x *g*) for 3 min after which the particles were washed three times with sterile deionized water. The microparticles were prepared fresh before every experiment and were used immediately. The efficiency of DnaK encapsulation was calculated as previously described (36). Thus, 200 mg (wet weight) of microparticles contained 300 μg of antigen or 1.5 μg antigen per mg microparticles.

### Axenic Sea Bass Larvae Production

European sea bass eggs that were produced by natural spawning and were pre-disinfected (20 ml l^−1^ 0.5% active iodine for 10 min) were obtained from Écloserie Marine (Gravelines, France). Upon arrival, the eggs were transferred to cylindro-conical tank and were acclimatized in UV-treated sea water for 4 h. For obtaining axenic sea bass larvae, the disinfection of eggs was performed following the protocol developed by Dierckens et al. (37). All larvae used in this study belonged to the same full-sibling family batch. Once disinfected, 600 eggs were incubated in 400 ml of filtered (0.45 μm; Sartorius, Vilvoorde, Belgium) autoclaved artificial sea water (Instant Ocean, United Pet Group, Virginia, US) and in presence of gentle filtered aeration (0.1 μm). During the incubation, salinity of 36 g l^−1^ and temperature of 16 ± 1°C was maintained. To sustain axenity, solutions containing rifampicin and ampicillin were simultaneously added in the sea water at a final concentration of 10 mg l^−1^. Axenity testing was performed by inoculating 30 of eggs as well as 1 ml of culture medium (collected from individual bottles) in 9 ml of sterile marine broth (Carl Roth GmbH Co., Karlsruhe, Germany) followed by incubation at 28°C for 96 h. Incubation bottles that were positive for bacterial growth were excluded from the experiment. All the handlings and procedures including hatching, stocking, and experiments, were carried out under laminar flow hood and in a temperature-regulated room (16 ± 1°C) with dim light (100 lux).

### DnaK Administration in Axenic Sea Bass Larvae and Challenge With *V. anguillarum*

**Figure 2:**
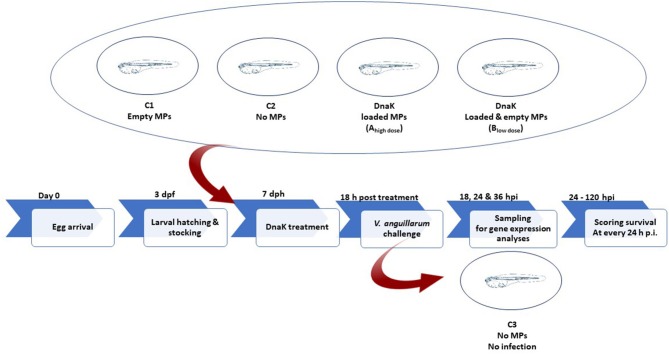
Schematic illustration of the experimental design.

For performing the experiment, axenic larvae on DAH6 were used. Larvae were stocked per 12 animals, in sterile screw cap vial containing 10 ml of filtered, autoclaved artificial sea water in which 10 mg l^−1^ rifampicin was added. The vials were placed on a rotor (4 rpm) with an axis tangential to the axis of the vials for providing aeration. Each group consisted out of 10 vials of 12 larvae (*n* = 120) (Table 1 and Figure 2).

**Table 1:** Experimental setup.

**Groups** **(*n* = 120)**	Administering MPs^*^ at day 7 after hatching	***V. anguillarum* infection 18 h post administering MPs**	**Number of larvae sampled p.i. for expression of immune-related genes**			**Daily recording of survival from 0 until 120 h p.i**.
			**18 h**	**24 h**	**36 h**	
A_high dose_	Loaded MPs^a^	Yes	36	36	36	Yes
B_low dose_	Loaded MPs^b^	Yes	36	36	36	Yes
C1	Empty MPs^c^	Yes	36	36	36	Yes
C2	No MPs	Yes	36	36	36	Yes
C3	No MPs	No	36	36	36	Yes

* *MPs = microparticles*.

a *1.0 mg MPs loaded with recombinant DnaK; representing 1.5 μg of DnaK*.

b *0.5 mg MPs loaded with recombinant DnaK + 0.5 mg empty MPs; representing 0.75 μg of DnaK*.

c *1.0 mg empty MPs*.

At DAH7, larvae had a mean body weight of 0.5 mg. At this stage, the animals of groups A_high dose_, B_low dose_ and C1 received a single dose of 1.0 mg alginate microparticles directly added into the sea water. Group A_high dose_ received 1.0 mg of loaded microparticles, representing 1.5 μg of DnaK. Group B_low dose_ received 0.5 mg empty microparticles and 0.5 mg loaded microparticles, representing 0.75 μg of DnaK. Group C1 received only 1 mg of empty microparticles. A control group (C2) in which animals received no microparticles was included in the study. Eighteen hours after administering the microparticles, groups A_high dose_, B_low dose_, C1 and C2 were experimentally infected (bath infection) with the virulent *V. anguillarum* strain HI-610 (serovar O2a, rifampicin resistant) using an infective dose of 10^5^ CFU. The bath infection model was previously validated during a pathogenesis study of a *V. anguillarum* infection in gnotobiotic sea bass larvae (38, 39). An extra control group (C3) in which the animals were neither treated nor challenged was included. Mortality in all groups was monitored in all vials by counting the living larvae (transparent and swimming) under a dissecting microscope at 18, 24 and 36 h p.i. At each of these time points, 36 larvae (whole-body larva sampling) per group (3 biological replicates of 12 larvae = 3 vials) were euthanized by over-anesthetization using methylsulfonatetricaine (MS-222, Sigma) for analyzing the expression of innate immune-related genes. Euthanized larvae were immediately snap frozen in liquid nitrogen and were subsequently stored at −80°C until analysis. The remaining 12 larvae per group were monitored till 120 h p.i. and the mortality in each group was recorded daily. At the end of the trial, all remaining larvae were euthanized by over-anesthetization. The experiment was carried out in accordance with the recommendations in the European Union Ethical Guidelines for the care of animals used for experimental and other scientific purposes (2010/63/EU). The trial was approved by the UGhent Ethical Committee for Animal Experiments (EC2014/147).

### Sampling and Reverse Transcription Real-Time PCR (RT-qPCR)

At 18, 24, and 36 h p.i., the total RNA of the larvae was extracted following the protocol described by Reyes-López et al. (40). Briefly, the larvae of each vial were sacrificed by over- anesthetization using sea water containing methylsulfonatetricaine (MS-222) (Sigma). The animals were snap-frozen in liquid nitrogen and were stored at −80°C for further analysis. For immune gene expression analysis, total RNA was extracted of triplicates of 12 pooled homogenized larvae from each group using the Total RNA Isolation Reagent (TRI Reagent® Molecular Research Center, Inc., Cincinnati, USA) (41). The quantity and quality of the RNA was tested using a NanoDrop 2000 spectrophotometry (NanoDrop Technologies Inc.,). The extracted RNA was then treated with RNase-free amplification grade DNase I (Thermo Scientific, Aalst, Belgium) following the manufacturer's protocol and confirmed to be DNA free by PCR for the ribosomal protein S18 (*rps18*) gene. One μg of total RNA was reverse transcribed (RevertAid™ H Minus first-strand cDNA synthesis kit; Thermo Scientific) following the manufacturer's protocol. RNA and cDNA samples were stored at −80 and −20°C, respectively.

Selected immune genes and the primers that were used for mRNA expression analysis are presented in Table 2. Amplified cDNA samples were subjected to RT-qPCR using a Rotor-Gene Q (Qiagen®) instrument with each reaction containing 15 μl master mix containing iQ™ SYBR® Green Supermix (Bio-Rad Laboratories), the optimum concentration of primer pair (Table 2) and 5 μl of diluted cDNA to a final volume of 20 μl. The RT-qPCR reaction was performed with one cycle of 95°C for 3 min and 40 cycles of 95°C for 15s, 57–58°C for 1 min and 72°C for 15 s. Each of the three biological replicates was analyzed in duplicate. Plasmids (pGEM®-T) that contained gene-specific insert were used as positive controls. non-reverse transcribed total RNA of larvae and ddH2O were used as negative controls. The endogenous *rps18* gene (housekeeping gene) was used as for normalization and the RT-qPCR efficiencies for each gene varied between 95 and 100%, allowing the use of the 2^−ΔΔCT^ method for calculation of relative gene expression (fold-changes) (45).

**Table 2:** Primer sequences used for RT-qPCR.

**Gene (protein)**	**Function**	**Accession no**.	Primer* sequence (5^**′**^-3^**′**^)	**Primer concentration (nM)**	**References**
*rps18* (RPS18)	Ribosomal protein S18 gene, house keeping gene	AM490061.1	F: AGGGTGTTGGCAGACGTTAC R: CTTCTGCCTGTTGAGGAACC	300	(42)
*tlr3* (TLR3)	Toll-like receptor, pathogen recognition receptor	AB675413.1	F:TGATCCAGCTAGTGAGACTAAGG R: TAGAGTCTGAAGTCTGGGCAGT	150	(43)
*tlr5m* (TLR5M)	Toll-like receptor, pathogen recognition receptor	JF266563.1	F: GGTCATCTTCAGCGGGATTGT R: GAAGACTGTGCATCGTCTC	150	(43)
*cas1* (caspase 1)	Caspase 1, protease	DQ198376.1	F: TATCATGTCGCACGGGAAACT R: TTTCTCTTCTCTCCAGCACCC	450	(43)
*il1β* (IL-1β)	Interleukin 1 beta, pro-inflammatory cytokine	AJ269472.1	F: ATCTGGAGGTGGTGGACAAA R: AGGGTGCTGATGTTCAAACC	300	(42)
*Tnfα* (TNFα)	Tumor necrosis factor alfa, pro-inflammatory cytokine	DQ070246.1	F: TGAGAGGTGTGAGGCGTTTTC R: GATTAGTGCTTCGGTTTGGCC	300	(43)
*Il10* (IL-10)	Interleukin 10, anti-inflammatory cytokine	DQ821114.1	F: CGACCAGCTCAAGAGTGATG R: AGAGGCTGCATGGTTTCTGT	300	(42)
*Mif* (MIF)	Macrophage migration inhibitory factor, pro-inflammatory cytokine	FN582353.1	F: CACTGAGGAGCTGGCGAAAG R: CACTACCTTTGGCTGAGAGGG	150	(43)
*cxcl8* (CXCL8)	CXC chemokine, chemotactic cytokine	AM490063.1	F: GTCTGAGAAGCCTGGGAGTG R: GCAATGGGAGTTAGCAGGAA	300	(42)
*cxcr4* (CXCR4)	Chemokine receptor 4, receptor for CXCL12, role in cell migration, proliferation and differentiation	FN687464.1	F: GCAAAGCACAGGGTCTTCAAA R: TACTGTGTTGGCATCTTTTCGG	150	(43)
*ccr9* (CCR9)	Chemokine receptor 9, receptor for CCL25 and role in migration, proliferation and apoptosis in leukocytes	FN665390.1	F: CTGATACTACCCCTGCCCTTTC R: TTCTCGTGTGCTGCTGTAACT	150	(43)
*cc1* (CC1)	CC chemokine 1, chemotactic cytokine	AM490065.1	F: CCTAACCGTGAATGTGTCCCA R: TCCGGCCAATGAAAACACCTA	150	(43)
*hpc (hepcidin)*	Antimicrobial peptide, iron regulation	DQ131605.1	F: GGAATCGTGGAAGATGCCGT R: CAGACACCACATCCGCTCAT	500	(40)
*dic (dicentracin)*	Antimicrobial peptide	AY303949.1	F: AGTGCGCCACGCTCTTTCTTGT R: TTGTGGATGGACTTGCCGACGTG	500	(44)

For the effect of *Vibrio* infection on the larval immune system, C2 (no MPs and challenged with *V. anguillarum*) gene expression profile was studied by calculating the immune gene expression relative to group C3 (no MPs and no *Vibrio*) as the calibrator by calculating ΔΔC_T_. For studying the effect of DnaK on immune gene expression in European sea bass larvae, mRNA expression analysis was performed relative to C1 (fed with MPs and challenged with *V. anguillarum*) as the calibrator by calculating ΔΔC_T_.

### Statistical Analysis

To determine significant differences between various treatments, survival data were subjected to logistic regression analysis by using GenStat 16 (VSN International, Hemel Hempstead, UK). Statistical analysis of gene expression was conducted using IBM SPSS Statistics for Windows, Version 21.0 (IBM Corp., Armonk, NY). For comparing the sea bass relative gene expression (fold-changes) between different treatments at each time point *t*-test analysis (comparisons between C2 and C3) and one-way ANOVA (comparison between C1, A_high dose_ and B_low dose_) were performed by setting the analyses at 95% confidence intervals (*P* < 0.05).

ANOVA test was followed by a *post-hoc* comparisons using Tukey HSD test and data for gene expression was log-transformed.

## Results

### Survival of Sea Bass Larvae

**Figure 3:**
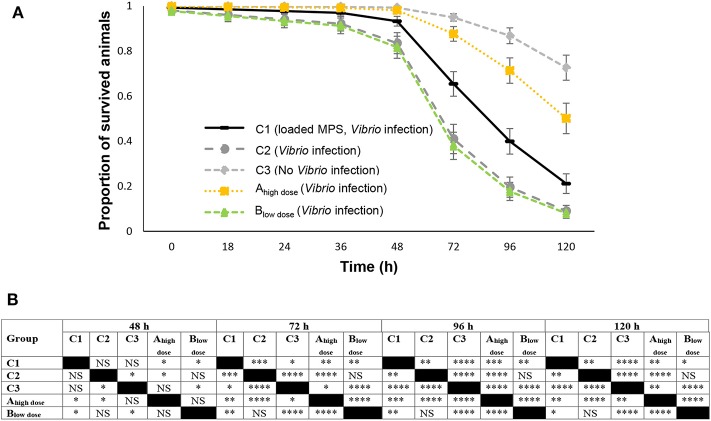
The proportion of surviving European sea bass larvae challenged with *V. anguillarum*. Animals of group C1 (1.0 mg of empty MPs), C2 (no MPs), A_high dose_ (1.0 mg of DnaK loaded MPs) or B_low dose_ (0.5 mg of empty MPs and 0.5 mg of DnaK loaded MPs) were challenged with 10^5^ CFU of *V. anguillarum*. Group C3 received no MPs and was not infected. Survival of the animals was scored before challenge (0 h p.i.) and at several time points p.i. till the end of the trial at 120 h p.i. **(A)** The proportion of survived animals (mean ± SD) is represented at each time point. **(B)** Statistical differences in live animals for various groups at different time points after infection with *V. anguillarum*. For all groups, the proportion of live fish larvae was significantly the same before 48 h p.i. Statistical differences in the proportion of survived animals for various treatment groups, following infection are presented from 48 h p.i. till the end of the experiment at 120 h p.i. An asterisks represents a significant difference between the respective groups [not significant (NS) (*P* > 0.05), significant *(*P* < 0.05), significant ** (*P* < 0.01), significant *** (*P* < 0.001) and significant ****(*P* < 0.0001)]. Comparison between a group and itself is marked in black.

During the first 36 h p.i., the proportion of survived gnotobiotic European sea bass larvae was statistically the same for all groups (Figure 3). At 48 h p.i., significant (*P* < 0.05) mortality occurred in all *V. anguillarum* infected groups, except for the group receiving the high dose (1.5 μg) of DnaK (group A_high dose_) (Figure 3). In the latter group, significant mortality, as compared to the non-infected control group C3, was only observed from 72 h p.i. onwards. Mortality in all infected groups, including group A_high dose_, augmented further toward the end of the experiment (Figure 3). At the end of the experiment (120 h p.i.), only 8% of the larvae of group C2 (no MP, infected), 7% of the larvae of group B_low dose_ and 21% of the larvae of group C1 (empty MP, infected) had survived the infection, showing the ineffectiveness of DnaK administration at a low dose of 0.75 μg. However, at the same time point, 50% of the larvae of group A_high dose_ were still alive and the proportion of survived animals in group A_high dose_ was statistically higher than in all other infected groups (Figure 3) (C1, C2, and B_low dose_), but lower than for group C3 (no MP, non-infected) which showed 73% survival. The survival results clearly demonstrated the beneficial effect of feeding European sea bass larvae with 1 mg of microparticles loaded with 1.5 μg DnaK (Figure 3). The administration of empty MPs (C1) also appeared to have some positive effect on survival (Figure 3) as at 120 h p.i., the survival rate in C1 was significantly higher than for group C2 (no MP, infected) and even group B_low dose_. However, at 120 h p.i., a significant difference (*P* < 0.01) of 41% was recorded between the survival in group C1 and group A_high dose_. Also, at h p.i. the proportion of survived animals in group A_high dose_ was significantly (*P* < 0.01) lower by 22%, as compared to the survival in the non-infected control group C3.

### Effect of a *V. anguillarum* Infection on Immune-Related Gene Transcription

**Figure 4:**
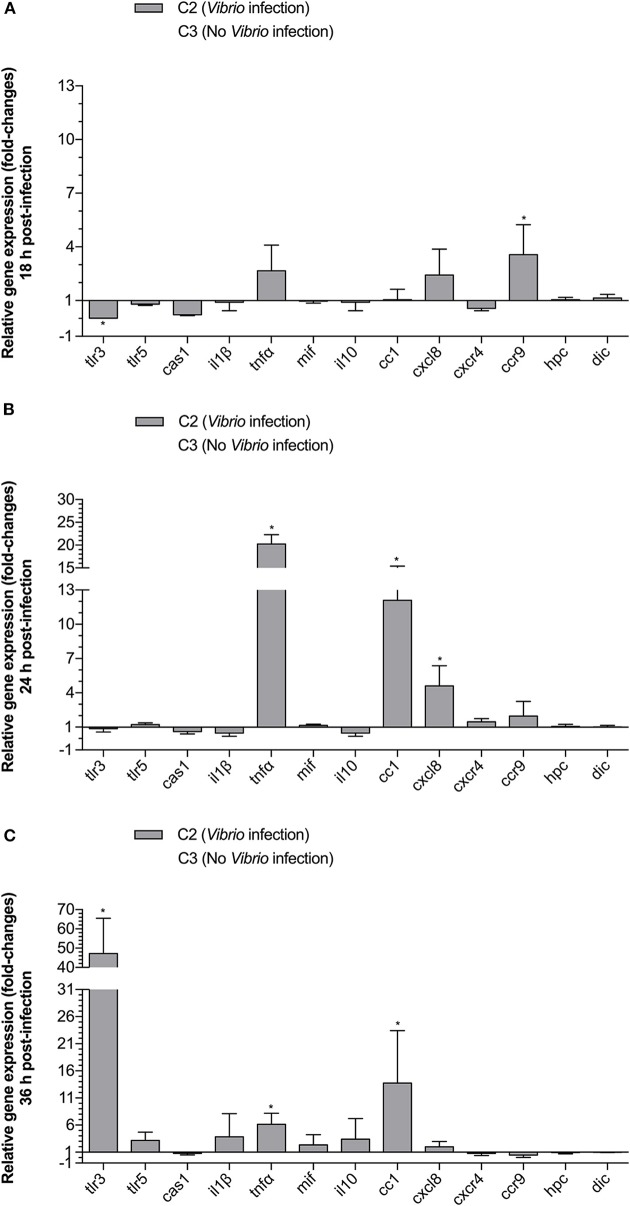
The effect of *Vibrio* challenge on relative expression of TLR, cytokine, chemokine and chemokine receptor genes in gnotobiotic sea bass larvae. Animals that were not fed with MPs were either cultured without challenge (C3) or were challenged with *V. anguillarum* at 10^5^ CFU (C2). Animals were sampled at 18 **(A)**, 24 **(B)**, and 36 **(C)** h p.i. Gene expression results for C2 are presented relative to the C3 values. Bars represent the standard deviation (SD) of the mean. An asterisks (*) represents a significant (*P* < 0.05) change in gene expression for C2 compared to C3.

In order to examine the effect of a *V. anguillarum* infection on immune-related gene expression early upon infection, we compared the *tlr3, tlr5m, cas1, il1*β, *tnf*α, *mif* , *il10, cc1, cxcl8, cxcr, ccr9, hpc*, and *dic* gene transcript levels for groups C2 (no MP, infected) and C3 (no MP, non-infected) at 18, 24, and 36 h p.i. The mean gene transcript levels ± SD for group C2 relative to group C3 are presented in Figure 4. At 18 h p.i., only *ccr9* (3.57-fold) was significantly upregulated. In addition, *tlr3* (0.02-fold) was significantly, downregulated at this time point. At 24 h p.i., *tnf*α (20.25-fold), *cc1* (12.11-fold), and *cxcl8* (4.60-fold) were significantly upregulated. At, 36 h p.i., *tlr3* (47.17-fold), *tnf*α (6.15-fold), and *cc1* (13.73-fold) were significantly upregulated.

### Effect of the Prophylactic Use of Recombinant *E. coli* DnaK on Immune-Related Gene Transcription Following a *V. anguillarum* Infection

**Figure 5:**
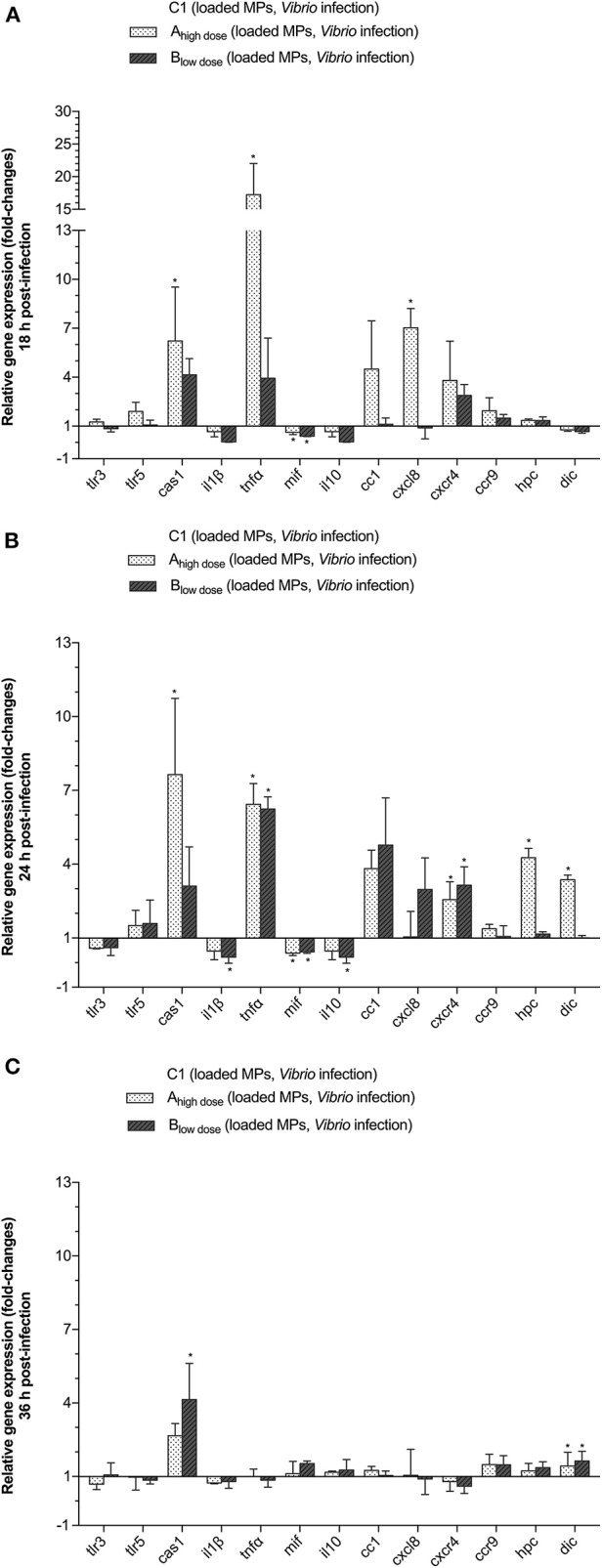
The effect of treating the animals with DnaK on the relative expression of TLR, cytokine, chemokine and chemokine receptor genes in gnotobiotic sea bass larvae. The larvae were divided into 3 different groups and were fed with either empty MPs (C1) or encapsulated DnaK at a high (A_high dose_) or low dose (B_low dose_) prior to infection with 10^5^ CFU of *V. anguillarum*. Animals were sampled at 18 **(A)**, 24 **(B)**, and 36 **(C)** h p.i. Gene expression results for the A_high dose_ and B_low dose_ groups are presented as fold-changes relative to C1. Bars represent the standard deviation (SD) of the mean. An asterisks (*) indicates a significant difference (*P* < 0.05) for the high (A_high dose_) or low dose (B_low dose_) group compared to C1.

We examined the prophylactic effect of DnaK administration prior to a *vibrio* infection, by studying the survival of the larvae as well as the expression of *tlr3, tlr5m, cas1, il1*β, *tnf*α, *mif* , *il10, cc1, cxcl8, cxcr, ccr9, hpc*, and *dic* genes in live animals sampled at 18, 24, and 36 h p.i. The mean gene transcript levels ± SD for groups A_high dose_ and B_low dose_ relative to group C1 (empty MP, infected) are presented in Figure 5. At 18 h p.i., *cas1* (6.2-fold), *tnf*α (17.24-fold) and *cxcl8* (7.04-fold) were significantly upregulated in group A_high dose_ as compared to the control group C1. By 24 h p.i., more genes became upregulated in group A_high dose_ as transcript levels for *cc1* (3.82-fold), *cxcr4* (2.56-fold), *hpc* (4.27-fold), and *dic* (3.37-fold) had also significantly increased as compared to the control group C1. For group A_high dose_, significant gene upregulation as compared to group C1 was no longer present at 36 h p.i. except for *dic* (1.43-fold). Significant downregulation of genes did not occur in group A_high dose_, except for the *mif* gene, which was slightly downregulated at 18 and 24 h p.i. as compared to the control group C1.

For group B_low dose_, at 18 h p.i., only *cas1* (4.14-fold) and *cxcr4* (2.88-fold) transcript levels were significantly increased compared to the control group C1. At that time, *il1*β (25-fold), *mif* (2.04-fold), and *il10* (25-fold) were significantly downregulated as compared to the control group C1. At 24 h p.i., only two genes mRNA were significantly upregulated in group B_low dose_, being *tnf*α (6.24-fold) and *cxcr4* (3.14-fold). Expression of *mif* (0.44-fold), *il10* (0.22-fold), and *il1B* (0.22) was, albeit slightly, downregulated at that time. Later on, at 36 h p.i. only *cas1* (4.14-fold) and *dic* (1.63-fold) were significantly upregulated as compared to the control group C1.

Regarding all genes under study, *cas1, cc1, cxcl8*, and *cxcr4* were significantly upregulated, albeit not at the same time, in both group A_high dose_ and group B_low dose_, as compared to the control group C1. In addition, for group A_high dose_, significant gene upregulation, as compared to the control group C1, was also observed for *tnf*α (18 h p.i.), *hpc* (24 h p.i.), and *dic* (24 h p.i.).

## Discussion

Industrial production of sea bass is still hampered by low and unpredictable survival of larvae and juvenile fish due to disease outbreaks such as infections caused by *Vibrio* spp. (46, 47). Vibriosis is a bottleneck for the expansion of sustainable sea bass aquaculture and causes high mortality especially during the larviculture. *Vibrio* spp. are actually opportunistic bacteria and therefore, vibriosis outbreak in larviculture is the result of the combination of various factors. For example, high larval density, accumulation of dead larvae and the introduction of bacteria through live food that can promote selection and growth of opportunistic bacteria, including *vibrio* (48). The latter might lead to *vibrio* infections and in case of sea bass larvae to *vibrio* replication in the skin, gills and gastrointestinal tract resulting in deadly septiceamic disease (49).

Stimulation of the innate immune system of sea bass larvae could perhaps protect them against *vibrio* infections. Therefore, the current study focuses on oral administration of a candidate immunostimulant, being *E. coli* HSP70, also known as DnaK, to induce a protective innate immune response in sea bass larvae against mortality caused by *V. anguillarum*. For this purpose, a previously generated experimental infection model in axenic sea bass larvae (37) was used exposing the animals to *vibrio* through the sea water (49).

Immunostimulants are commercially available for fish farming. Seaweed-based (AQUAVAC® Ergosan™; MSD Animal Health, New Jersey, USA), yeast β-glucan-based (MacroGard® Biorigin, Antwerp, Belgium and Bio-Mos® Alltech Inc, USA) and *Saccharomyces cerevisiae* beta-glucans and mannanoligosaccharides-based (ExcelMOS®, Global Nutritech; VA, USA) immunostimulants have been successfully applied in juvenile rainbow trout (*O. mykiss*), juvenile/adult sea bass (*D. labrax*), juvenile carp (*Cyprinus carpio*) and seabream (*S. aurata*) broodstock (50–55). In fish farms, these commercial immunostimulants are being incorporated in the diet. The application of other immunostimulants such as vitamins (i.e., vitamin C and E), microorganisms (i.e., probiotic bacteria), prebiotics (Bio-Mos®), hormones (i.e., thyroxine), biologically active compounds (i.e., antimicrobial peptides, lectins, bovine lactoferrin), medicinal herbs [i.e., *Astragalus membranaceus* and *Lonicera japonica* (honeysuckle) extracts], amino acids (i.e., DL-arginine), poly-β-hydroxybutyrate and organic pigments (i.e., carotenoid and astaxanthin) has also been studied in various aquaculture species (44, 56–59). However, data from application of immunostimulants in fish larvae are limited [reviewed by (18, 60)]. Nevertheless, the existing data point in the direction of considerable benefit and little detrimental effect to the developing animal (44).

Microparticles (MPs) are delivery systems used for the administration of immunostimulants in a controlled manner (61). Delivery of immunostimulants in fish using different types of microparticles has been described. Microparticles out of a synthetic amino acid being poly(D,L-lactide-co-glycolide) or PLGA or out of natural polymers such as calcium phosphate particles, chitosan particles, liposomes, and alginate particles have been used for targeting the immune system of fish (62, 63). Inorganic materials such as carbon nanotubes have also been used as a delivery system. However, they are not biodegradable (64). Natural polymers are most widely used due to their bioactivity, biocompatibility, chemical stability, low toxicity, susceptibility to enzymatic degradation and low production costs (65). One of the commonly used natural polymers is alginate. Alginate MPs can be prepared by extruding a solution of sodium alginate containing proteins as a droplet into a divalent cross-linking solution such as a Ca^2+^ solution. Gelation and cross-linking of alginate polymers is than mainly achieved by the exchange of sodium ions from the guluronic acids of the alginate with the divalent cations of the cross linking solution. A characteristic “egg-box” structure is formed by the stacking of these guluronic groups (66). By selecting the alginate type and the formulation conditions it is possible to control the characteristics of the alginate MPs delivering the encapsulated immunostimulant in a controlled release manner.

In larviculture, encapsulated immunostimulants in alginate MPs can easily be introduced into the water. This action does not provoke stress to the fish larvae. Microparticles of the correct size can be designed to ensure the uptake of the particles by marine fish larvae. Formerly, we evaluated MP uptake in axenic European sea bass at day after hatching 7 (DAH7; the mouth opening begins between DAH4 and DAH5) using fluorescent MPs. We observed that smaller MPs (<20 μm) were taken up passively, thus unintentionally, through the natural drinking activity, which was actually rather low (4.1 ± 0.1 nl h^−1^ larva^−1^) in sea bass larvae. Larger MPs (45 to 80 μm) were taken up actively as they were more easily noticed by the larvae and probably regarded as prey (33). Alginate MPs with a median size of 83.19 ± 4.73 μm were optimal for administration to sea bass larvae at DAH7, as 2 h after feeding a mean of 81.9 ± 9.0% of the larvae had ingested the MPs and by 48 h after feeding 100% of the larvae showed an accumulation of MPs along the midgut. The MPs disintegrated in the hindgut releasing their content locally. So, in the correct study, we created an alginate carrier matrix for the recombinant DnaK for oral delivery to axenic sea bass larvae at DAH7. The encapsulation efficiency was 19.7% which was the same as described by Yaacob et al. (33). The carrier matrix protected the DnaK from the external environment as it was stable in sea water (33) and provided localized delivery to the gastrointestinal tract of fish larvae.

In the current study, we first examined the expression of innate immune-related genes during a *V. anguillarum* infection in axenic sea bass larvae, comparing group C2 (no MP, infected) to C3 (no MP, non-infected). The expression of sensor (*tlr3, tlr5m, cxcr4, ccr9*), regulatory (*il10, cas1, il1*β, *tnf*α, *mif1, cxcl8*) and effector (*hpc, dic*) genes was studied early upon infection as the first time points of infection are critical for fish immunity (67). Previously, we demonstrated the expression of all sensor and regulatory genes under study in axenic sea bass larvae (43). The expression of *hpc* and *dic* has been demonstrated by others in axenic and non-axenic sea bass larvae, respectively (44, 67). Possible *Vibrio* (Gram-negative bacterium with a flagellum) pathogen associated molecular patterns (PAMPs) that might stimulate the pathogen recognition receptors (PRRs) of fish are flagellin (TLR5), peptidoglycan (TLR1 and 2, and NOD1 and NOD2), lipoproteins (TLR1 and 2), lipo-arabinomannan (TLR1 and 2) and glycosyl-fosfatidylinositol (TLR1 and 2). Thus, TLR5M is expressed in European sea bass larvae (43), but we do not know if the other PRRs for *Vibrio* are present at these early live stages and if they are already expressed on innate immune cells and/or mucosal epithelial cells in the skin, the gills and the gut, which are the primary replication sites for *Vibrio*. At present, we noticed the expression of *tlr5m* (receptor for bacterial flagellin) in larvae but transcript levels were statistically the same for all groups. We noticed a significant upregulation for *tlr3* in group C3, but we have no explanation for this observation. Actually, only few other immune-related genes were upregulated following infection. The latter is in contradiction with our previous results using the same experimental infection model, the same *V. anguillarum* strain and the same infective dose (43). During the latter study, *V. anguillarum* highly upregulated the expression of all genes under study (the same gene panel as currently with the exception of *hpc* and *dic*) except for *cxcr4* and *ccr9*. Significant mortality occurred also later (from 72 hp.i. onwards) than in the current study. Perhaps, animals used in the present study, which came from another broodstock, were less robust and/or less immunocompetent, as at the end of the trial (120 h p.i.) only 73 ± 0.05% of the animals in group C3 (no MP, non-infected) were still alive, while in our former study 96.67 ± 3.33% were still alive at 120 h p.i. Indeed, we need to consider that all the individuals in the present and former study were full-sibling (limited genetic variability), but they originated from another broodstock. In Atlantic salmon (*Salmo salar*), it has been demonstrated that the same pathogen can differentially affect the survival of different full-sibling groups and that this is due to differences in their ability to mount an effective immune response (67). Differences in survival rates have also been observed by Dierckens et al. (37), performing two independent *V anguillarum* (strain HI-610 at 10^5^ CFU) challenge trials in axenic European sea bass larvae.

In the current study, we showed that 1 mg alginate microparticles, containing in total 1.5 μg (group A_high dose_) recombinant *E. coli* HSP70 (DnaK) could be added to the sea water, resulting in the uptake of microparticles and significantly augmented transcription levels for immune-related sensor (*cxcr4*), regulatory (*cas1, tnf*α*, cc1*, and *cxcl8*) and effector (*hpc, dic*) genes. Thus, significantly elevated transcription levels for sensor, regulatory and effector genes were correlated with increased resistance of the larvae to a lethal infection with *V. anguillarum*. Whether the augmented transcription of *tnf*α*, cc1* and *cxcl8* in group A_high dose_ was due to DnaK is not certain, as these genes were also upregulated by the *Vibrio* infection itself. However, in group A_high dose_, significantly augmented expression levels for *tnf*α and *cxcl8* were observed earlier (18 h p.i.) upon infection as compared to group C2 (no MP, infected). Group B_low dose_ was not significantly protected. This might be due to the fact that only sensor (*cxcr4*) and regulatory (*cas1, cc1*, and *cxcl8*) genes were significantly upregulated and not the effector genes *hpc* and *dic*, at least not at 18, 24, and 36 h p.i. Strangely, the administration of empty MPs also seemed to have some positive effect on survival following infection as at 120 h p.i., the survival rate in group C1 was significantly higher than in group C2 (no MP, infected). This is possibly due to the fact that animals received no larval fish food during this trial (to ensure axenic conditions), but alginate MPs can also be used as food, rendering the larvae of group C1 more robust than the ones from group C2. At 120 h p.i., the survival rate in group C1 was also significantly higher than in group B_low dose_. Here, the metabolic cost of an albeit inefficient prophylactic strategy (too low dose of DnaK) might be the reason for the observed difference in survival.

Both *cxcr4* and *cas1* were significantly upregulated by DnaK administration, and in our study, not by *Vibrio*. CXCR4 is a master regulator of innate immune responses. CXCR4 signaling is important for cell migration, proliferation and differentiation (68). *Cas1* mRNA levels were upregulated, but it was not accompanied or followed by an upregulation of *il1*β mRNA levels. *Cas1* upreglation could have played a role in pyroptosis, a gasdermin (GSDM)-dependent inflammatory type of programmed cell death (69). Different from mammals, which have a panel of pyroptotic GSDM members (e.g., GSDMA-E), teleosts only possess GSDME. Jiang et al. (69) found that teleost GSDME exerted pyroptotic activity and bactericidal activities through its N-terminal domain. GSDME is specifically and highly efficiently cleaved by teleost caspase 1, releasing the N-terminal domain. The N-terminal domain exhibits lipid-binding and pore forming capacity, which, when recruited to the cell membrane, leads to cell death and massive release of proinflammatory cellular content. The N-terminal domain of GSDME was also lethal to *E. coli*. Thus, elevated *cas1* expression could have contributed in this way to protection against *V. anguillarum*. However, this needs to be examined in more detail.

It is clear from our results that augmented expression levels for *hpc* and *dic* contributed to the observed partial protection in the infected groups. Recently, both *hpc* and *dic* mRNA levels and hepcidin and dicentracin peptides have been found in European sea bass eggs and throughout larval development (70). Also, upregulation of *hpc* was shown to be directly related to reduced mortality in European sea bass challenged with *V. anguillarum* HI-610 (bath 10^8^ CFU) at DAH5 (40). In addition, it was shown that synthetic hepcidin of fish could induce significant protection in sea bass challenged with *V. anguillarum* (71). Meloni et al. (72) described the importance of dicentracin in protecting juvenile European sea bass against *V. anguillarum* and more recently, Franke et al. (44) suggested that poly-*B*-hydroxybutyrate (PHB)-induced *dic* expression might play a role in protection of European sea bass postlarvae (DAH 28–38) against vibriosis. Similar results were obtained in axenic Nile tilapia (*Oreochromis niloticus*) larvae (73).

In response to pathogens, fish cells secrete hepcidin and/or dicentracin amongst a number of other AMPs which possess different antimicrobial activities such as formation of transmembrane pores, hindering synthesis of cell-wall and inhibiting cytoplasmic membrane septum formation thus disrupting membrane structure. In addition, they can also deactivate enzymes and inhibit the synthesis of proteins as well as nucleic acids [reviewed by (74)]. Hepcidin has a dual function, it exerts antimicrobial activities but if also plays a role in iron regulation removing iron from the bloodstream and rendering it unavailable for bacterial growth. Hepcidin can bind and interfere with the outer membrane of bacteria leading to its destruction and eventual death of the bacteria. Furthermore, hepcidin can also destroy bacteria through interaction and hydrolysis of bacterial DNA (75). Dicentracin belongs to the family of piscidins. Fish piscidins are stored in granules of phagocytic granulocytes and are delivered to pathogen-containing phagosomes upon phagocytosis to kill the pathogens (76).

## Conclusions

Our study shows that *V. anguillarum* induces an immune response in axenic sea bass larvae but that the innate immune response was incapable to protect the larvae against deadly septicemic disease. In addition, we showed that administration of alginate encapsulated recombinant *E. coli* HSP70, also known as DnaK, to axenic European sea bass larvae at DAH7 resulted in a significant, DnaK dose dependent, upreglation of immune sensor, regulatory and effector genes. Significant upregulation of *cxcr4, cas1* and especially of *hep* and *dic* was correlated with significant higher survival rates in *V. anguillarum* infected larvae. In the future, recombinant DnaK might perhaps be used as a novel immunostimulant in sea bass larviculture. However, our study showed that there is still room for improvement as mortality still occurred in the DnaK treated larvae. A higher DnaK dose and/or repeated administrations instead of a single dose could perhaps further improve this immunostimulatory strategy.

## Data Availability Statement

All datasets generated for this study are included in the article/supplementary material.

## Ethics Statement

The experiment was carried out in accordance with the recommendations in the European Union Ethical Guidelines for the care of animals used for experimental and other scientific purposes (2010/63/EU). The trial was approved by the UGhent Ethical Committee for Animal Experiments (EC2014/147).

## Author Contributions

EY and PN wrote the paper and PN assisted in reviewing the results. BD provided scientific advice for the preparation of the alginate microparticles. KD, PB, and DV designed and supervised the study, assisted in drafting the paper and critically reviewed the paper. AB provided the seabass larvae for the experiment.

### Conflict of Interest

AB is employed by the company Écloserie Marine de Gravelines. The remaining authors declare that the research was conducted in the absence of any commercial or financial relationships that could be construed as a potential conflict of interest.
